# Sleep deprivation causes gut dysbiosis impacting on systemic metabolomics leading to premature ovarian insufficiency in adolescent mice

**DOI:** 10.7150/thno.95197

**Published:** 2024-06-17

**Authors:** Jiamao Yan, Xiaoyuan Zhang, Kexin Zhu, Mubin Yu, Qingchun Liu, Massimo De Felici, Teng Zhang, Junjie Wang, Wei Shen

**Affiliations:** 1 College of Life Sciences, Qingdao Agricultural University, Qingdao 266109, China.; 2 State Key Laboratory of Reproductive Regulation and Breeding of Grassland Livestock, College of Life Sciences, Inner Mongolia University, Hohhot 010021, China.; 3 Department of Biomedicine and Prevention, University of Rome Tor Vergata, Rome 00133, Italy.

**Keywords:** Sleep deprivation, Premature ovarian insufficiency, Gut microbiota, Niacinamide metabolism, Mouse

## Abstract

**Rationale:** Currently, there are occasional reports of health problems caused by sleep deprivation (SD). However, to date, there remains a lack of in-depth research regarding the effects of SD on the growth and development of oocytes in females. The present work aimed to investigate whether SD influences ovarian folliculogenesis in adolescent female mice.

**Methods:** Using a dedicated device, SD conditions were established in 3-week old female mice (a critical stage of follicular development) for 6 weeks and gut microbiota and systemic metabolomics were analyzed. Analyses were related to parameters of folliculogenesis and reproductive performance of SD females.

**Results:** We found that the gut microbiota and systemic metabolomics were severely altered in SD females and that these were associated with parameters of premature ovarian insufficiency (POI). These included increased granulosa cell apoptosis, reduced numbers of primordial follicles (PmFs), correlation with decreased AMH, E2, and increased LH in blood serum, and a parallel increased number of growing follicles and changes in protein expression compatible with PmF activation. SD also reduced oocyte maturation and reproductive performance. Notably, fecal microbial transplantation from SD females into normal females induced POI parameters in the latter while niacinamide (NAM) supplementation alleviated such symptoms in SD females.

**Conclusion:** Gut microbiota and alterations in systemic metabolomics caused by SD induced POI features in juvenile females that could be counteracted with NAM supplementation.

## Introduction

Sleep plays a vital role in human physical and mental health. A lack of high-quality sleep weakens bodily defenses against disease and medical conditions. Numerous studies have highlighted the negative health effects of acute and chronic sleep deprivation (SD), including cognitive developmental impairment, decline of creative ability, and disorders of the gut microbiota, endocrine system, and immune function, thereby inducing various physical and mental diseases and even death 1-9. Meanwhile, reproductive disfunctions are also connected with SD. In animal models, the impaired secretion of sex hormones caused by SD is reported to decrease testosterone levels, reduced sperm motility, and cause apoptosis of Leydig cells in the testis 10. In female mice, SD reduces the expression of uterine circadian genes and affects early pregnancy outcomes through inhibition of IL-6-dependent endometrium decidualization 7. Furthermore, pregnancy and perinatal disorders are reported to be associated with insomnia in humans 11. Moreover, sleep disturbances are increasingly recognized as important factors in women's reproductive health, particularly the menstrual cycle, pregnancy, and menopause 12, 13. However, the effects of sleep deprivation on fertility in female animals is largely unknown.

Premature ovarian insufficiency (POI) is an infertility disorder characterized by cessation of ovarian function that affects 3.7% of women before the age of 40 14, 15. POI is a highly heterogeneous disease that may be a result of the following: a small pool of primordial follicles, impaired follicular development, follicle dysfunction, or premature follicle depletion due to accelerated atresia 16, 17. The etiology of POI is diverse, and may be of genetic, iatrogenic, autoimmune, infectious environmental, or metabolic origin; these can be grouped into two categories: genetic and non-genetic causes 18. For genetic aspects of POI, approximately 110 causative genes have been identified based on either a physiological role in ovaries or the phenotype of mutant mouse models. These include genes involved in DNA damage repair (*Mcm9*, *Spidr*), follicle development and maturation (*Gdf9*, *Bmp15*, *Wt1*, *Cpeb1*, and *Foxl2*), ovulation (*Alox12*), and hormones and receptors (*Amh*, *Esr2*, *Fshr*, and *Lhcgr*) 14, 17. Among non-genetic causes for POI, the effects of metabolic disorders cannot be ignored. Accordingly, mutation of the GALT gene would impair the function of galactose-1-phosphate uridylotransferase, and trigger a metabolic change in galactose, leading to POI 19. In addition, 17-hydroxylase deficiency can block synthesis of all 19-carbon steroids, causing POI 20. In addition, gut microbiota dysbiosis, such as disturbance of core microbiota (*Bacteroides* and *Lachnoclostridium*) or an increase of opportunistic pathogenic bacteria, has been suggested to be connected with POI 21, 22. Therefore, the pathogenesis of POI is relatively complex, and understanding its origins will be conducive to the clinical treatment of POI patients.

Recent studies indicate that sleep deprivation and disruption are associated with impaired reproductive function and poor reproductive outcomes in women 23. Furthermore, hypothalamic-pituitary-adrenal axis (HPA) activation and suppression or augmentation of reproductive hormones and alteration of gut microbiota, are important ways in which, SD can affect female reproduction and fertility 12, 24. Importantly, the gut microbiota creates a bridge between external environmental stimuli and host metabolism since it participates in the transformation and absorption of nutrients, and directly or indirectly impacts on a variety of tissue and organ functions. Studies have shown that the intestinal microbiota plays an important role in the occurrence and development of reproductive endocrine diseases, and is closely related to host health 25-27. For example, studies have found that gut microbiome imbalance in a metabolic syndrome model significantly lowers bile acid levels and causes abnormal metabolism of vitamin A, which in turn affects testicular cells through circulation and impaired spermatogenesis 28. Recently, it has been suggested that the gut microbiota is associated with metabolic disorders in women with PCOS, and that its modification alters bile acid metabolism and/or increases IL-22 levels which may be of value for the treatment of PCOS 29. In addition, dysregulation of the gut flora leading to increased beta-glucuronidase activity, has been reported to elevate estrogen levels, leading to reproductive diseases and cancer 30.

In the present study, we established a mouse model of SD in adolescent female mice and analyzed the effects of SD on ovarian follicle dynamics and reproductive performance from the perspective of gut microbiota and systemic metabolism changes induced under this condition. Results indicated that SD was capable of changing gut microbiota and systemic metabolism, and these reduced female reproductive performance; this can be alleviated by dietary supplementation of NAM metabolite.

## Methods

### Animal treatment and sample collection

Three-week-old C57BL/6J female mice were purchased from Jinan Pengyue Experimental Animal Breeding Center (Jinan, China). They were housed under a standard 12-h light/12-h dark cycle at 23±1°C, 50%-60% humidity, and ad libitum food and water consumption for 3 days before experimental treatment. All procedures were approved by the Animal Ethics Committee of Qingdao Agricultural University (approval No. 2022-0021).

The mouse model of sleep deprivation (SD) was established for 20 h per day for 6 weeks by using the dedicated device KW-BD, purchased from NJKEW Biotechnology Co., Ltd (Nanjing, China). Briefly, a disturbance bar at the bottom of the SD device, rotates and slides at a constant frequency with a working speed of 1.0 r/min, moving under the mouse's body, thereby preventing sleep (for a detailed description of the instrument, see http://www.kw689.com/product/yd/info128.html). SD time was set from 18:00 to 14:00 h of the next day. Animals were weighed every 2 days and at the end of SD treatment blood samples were collected from the eye and the mice were sacrificed by neck dislocation. Ovaries, small intestine, and small intestinal contents (gut microbiota) were rapidly collected in a sterile environment, snap frozen in liquid nitrogen, and stored at -80°C for further analysis.

### Fecal microbiota transplantation

Fecal microbiota transplantation (FMT) was performed as previously described 31, 32. Briefly, small intestinal content was collected and thoroughly mixed with 20% glycerol at a ratio of 1:1, quick frozen in liquid nitrogen, and stored at -80°C. Before use, the sample was diluted with saline solution to a working concentration of 50 mg/ml and filtered through a 70 μm cell sieve. Each recipient mouse received 100 μL/d filtrate by oral gavage for 2 weeks followed by regular feeding for 3 weeks.

### Niacinamide supplementation

Niacinamide (NAM) powder was stored and sealed at -20°C, to ensure the consistency of experimental results. NAM was diluted with water to the required concentration (100, 200, 400 mg/ml) before use, so that similar volumes were administered to mice daily at a working dose of 100, 200, or 400 mg/kg body weight. Intragastric NAM was administered for 6 weeks under SD conditions.

### Histology and immunohistochemistry

The procedures for ovarian histology and immunohistochemistry (IHC) have been previously described in detail 33. Briefly, ovaries were fixed overnight in paraformaldehyde (Solarbio, P1110), flushed with water, dehydrated with graded alcohols, and transferred into xylene before paraffin embedding. Ovaries were serially sectioned to a thickness of 5 µm and sections were placed onto slides for drying on a heated plate at 42°C. Samples were then left in an incubator at 60°C for 2 h and treated with xylene twice for 15 min each at room temperature (RT). After rehydration in graded alcohols, the sections were washed in PBS for 10 min, placed in citric acid antigen retrieval solution at 96°C for 10 min, and after cooling, sequentially treated with tris-buffered saline (TBS) and TBST (TBS with Tween-20) for 5 min. Samples were then treated with 3% H_2_O_2_ to remove endogenous peroxidase activity, reacted with BDT (TBS containing 3% BSA and 10% goat serum) at RT for 30 min, and incubated in primary antibodies overnight at 4°C. The next day, slides were incubated with a peroxidase-conjugated secondary antibody (Beyotime, A0208, Shanghai, China) at 37°C for 45 min. Finally, the samples were sequentially stained using a DAB kit (ZSGB-BIO, ZLI-9017, Beijing, China) and hematoxylin (Sangon Biotech, E607317), sealed with neutral resin, and observed under an Olympus BX51 microscope.

Starting from the middle section of the ovary, additional sections were selected every 15-20, both forward and backward, to give a total of 9 sections/ovary that were scored. Follicles were classified as i) primordial if they contained an oocyte surrounded by a single layer of flattened granulosa cells; ii) primary if they contained an oocyte surrounded by a single layer of flattened and cuboidal granulosa cells; iii) secondary if they contained an oocyte surrounded by 2 to 3 layers of cuboidal granulosa cells and no antral space; and iv) antral if they contained an oocyte surrounded by >3 layers of granulosa cells and an antral space. To avoid double-counting, only follicles containing oocytes with visible nuclei were considered 34.

### Small intestine histology and immunofluorescence staining

Similar to ovarian tissues, small intestine tissues were fixed, embedded, and then continuously sectioned at 7 μm thickness. After treatment with xylene and alcohol, the samples were stained with hematoxylin and eosin (Solarbio, G1100) for histological observation.

For small intestine tissue immunofluorescence (IF) staining, slides carrying sections were sequentially immersed in xylene, alcohol, PBS, and sodium citrate buffers before staining. After cooling, the slides were reacted with BDT at RT for 30 min; subsequently, they were incubated with the primary antibody at 4°C overnight. The next day, the slides were incubated with secondary antibodies in a dark room at 37°C for 45 min, and then the nuclei were stained with Hoechst 33342 at RT for 5 min. Finally, each slide was sealed with an anti-fluorescence attenuation quencher. The slides were then viewed under a fluorescence microscope.

### ELISA

The levels of NAM, FSH, LH, AMH, and E2 in blood serum samples were measured using dedicated enzyme-linked immunosorbent assay (ELISA) kits: JM-11773M2, JM-02838M2, JM-11607M2 (Jingmei Biological Technology, Jiangsu, China), and JB1334-Mu, JB973-Mu (Jinma Biotechnology, Shanghai, China), respectively, according to the manufacturer's instructions.

### Western blotting

Ovarian tissues were lysed in appropriate amounts of RIPA (Beyotime, P0013C) and sodium dodecyl sulfate (SDS) buffers. The extracted proteins were denatured by boiling the samples for 5 min and then stored at -20°C. Electrophoresis was conducted using a BioRad-PowerPacBasic electrophoresis apparatus (BIO-RAD, Taiwan, China). Proteins were transferred from the gel to a poly (vinylidene fluoride) membrane (PVDF, Millipore, ISEQ00010) at 200 mA using the wet-transfer method. After blocking for 4 h in TBST containing 5% BSA, the membranes were incubated with primary antibodies overnight at 4°C. The next morning, after washing with TBST, they were incubated with horseradish peroxidase-conjugated secondary antibodies for 90 min at RT and target protein bands were detected using a BeyoECL Plus kit (Beyotime, P0018). Proteins were quantified using ImageJ software. The intensities of the protein bands were normalized to GAPDH, and the relative amounts of the target proteins compared to that of the control. The antibody information used is listed in Table S1.

### TUNEL staining

Paraffin ovary sections were dewaxed with xylene and rehydrated with graded alcohols. TUNEL staining was conducted using a TUNEL BrightGreen Apoptosis Detection Kit (Vazyme, A113-02, Nanjing, China), following the manufacturer's instructions and counterstained with Hoechst 33342 (Beyotime, C1022) for 5 min. Finally, after the addition of anti-fluorescence quencher, the slides were sealed and stored in the dark at 4°C or directly observed under a microscope.

### *In vitro* maturation and meiotic spindle staining of the oocytes

Fully-grown germinal vesicle (GV) oocytes were collected and cultured following standard protocols as described in previous papers 33. The number of oocytes undergoing germinal vesicle break-down (GVBD) and first polar body extrusion (PBE) were counted after 4-6 h and 12-16 h, respectively. In order to visualize the meiotic spindle, oocytes were fixed in 4% paraformaldehyde for 30 min at RT, and permeabilizated in PBS supplemented with 0.5% Triton X-100 for 20 min. After blocking in PBS supplemented with 1% BSA for 1 h, the oocytes were incubated with α-tubulin antibody (Invitrogen, 322588) for 2 h and finally rinsed in washing buffer (PBS plus 0.1% Tween 20 and 0.01% Triton X-100) and stained with Hoechst 33342 for 15 min at RT. A laser-scanning confocal microscope (Leica TCS SP5 II, Mannheim, Germany) was used to observe and capture representative images.

### Real-Time Quantitative PCR

Total RNA was extracted from ovarian tissue according to the instructions of a SPARKeasy Improved Tissue/Cell RNA Extraction Kit (Sparkjade, AC0202, Shandong, China), followed by reverse transcription using SPARKscript II RT Plus (Sparkjade, AG0304, Shandong, China). Real Time quantitative PCRs (RT-qPCRs) were conducted using a SYBR Premix Ex Taq^TM^ fluorescent dye and CFX96 Real-Time PCR instruments (BioRad-CFX96, USA). The primers used are listed in the Table S2.

### Single cell RNA sequencing (scRNA-seq)

Oocytes were individually lysed, and cDNA synthesis and amplification performed following the SMART-seq2 protocol. The cDNA libraries were then checked on a Bioanalyzer instrument (Agilent), and the passed samples were finally chosen for sequencing. Sequence libraries were constructed using Annoroad Gene Technology Corporation (Beijing, China), and were sequenced by Illumina NovaSeq 6000 using the 150 bp paired-end strategy. Oocyte samples from 3 different ovaries were mixed and treated as one replicate, with 2 replicates per group for scRNA-seq. The scRNA-seq data were treated with FastQC (v0.11.8) for quality control and then aligned with the reference genome sequence 35. FPKM (fragment per kilobase of transcript per million mapped reads) values were calculated to quantify gene expression abundance. Differential expression RNA analysis was performed using DESeq2 software comparing 2 different groups 36. Differentially expressed genes (DEGs) were identified with a parameter of false discovery rate (FDR) <0.05 and absolute fold change (FC) ≥2. Pathway enrichment analysis was performed using Metascape 37.

### RNA sequencing (RNA-seq)

Total RNA was extracted from ovaries according to the manufacturer's instructions. High quality RNA was used for cDNA library construction and Illumina sequencing by Annoroad Gene Technology Corporation (Beijing, China). Firstly, FastQC (v0.11.8) was used to check the quality of sequencing raw data. FASTP (v0.23.1) software was used to remove low-quality data, including joint contaminated reads and low-quality reads. High-quality data were aligned to a reference genome using STAR (v2.7.0f) software, and gene counts were calculated using FeatureCounts (v1.6.3) software.

### 16S rRNA sequencing and analysis

16S rRNA sequencing analysis was performed with 15 samples in each group (30 in total). First, genomic DNA was extracted from the small intestine contents. The amplified V3-V4 region sequences were pooled and purified using a Qiagen gel extraction kit. Sequencing libraries were generated using TruSeq® DNA PCR-Free Sample Preparation Kit (Illumina, USA) following manufacturer's instructions. The library was sequenced using the Illumina NovaSeq platform (Novogene, Beijing, China) and 250 bp paired-end reads were generated. Subsequently, we used QIIME2 software (v2020.6) to merge paired ends and perform quality control; the DADA2 amplicon algorithm was used to remove sequencing noise, delete chimera sequences; and amplicon sequence variants (ASVs) were selected to generate signatures of each sample (feature table). Sequences with a similarity ≥97% were assigned to the same operational taxonomic unit (OTU). Based on bioinformatics, the alpha diversity indices of samples in groups were analyzed (including ACE, Chao1, Shannon, and Simpson). ANOSIM similarity analysis using the Bray-Curtis algorithm was used to determine differences between groups. Representative species were determined using an online tool (https://huttenhower.sph.harvard.edu/galaxy/) for linear discriminant analysis effect size (LEfSe) analysis.

### Non-targeted metabolomics

Non-targeted metabolomics were performed in collaboration with Shanghai Applied Protein Technology Co., Ltd (Shanghai, China). First, an appropriate amount of serum sample was added to pre-cooled methanol/acetonitrile/water solution (2:2:1, v/v), and vortexed, and mixed; it was then exposed to ultrasound at low temperature for 30 min, subsequently the centrifugal supernatant was taken and dried in a vacuum. During mass spectrometry analysis, 100 μL of acetonitrile aqueous solution (acetonitrile: water =1:1, v/v) was added for resolution. After vortex centrifugation, the supernatant was taken for sample analysis. Then the samples were separated by Agilent 1290 Infinity LC ultra-high performance liquid chromatography (UHPLC) HILIC column: column temperature was 25°C; flow rate was 0.5 mL/min; injection volume was 2 μL. After separation, mass spectrometry was performed with a Triple TOF 6600 mass spectrometer (AB SCIEX) using positive and negative ion modes of electrospray ionization (ESI), respectively. The raw data were converted into files in mzXML format by ProteoWizard, and then XCMS software was used for peak alignment, retention time correction, and peak area extraction. Structures of metabolites in biological samples were identified by matching with information of metabolite retention time, molecular mass (molecular mass error is less than 10 ppm), second-order fragmentation spectrum, and collision energy 38. According to the results of metabolite identification, the quality of experimental data was evaluated, and then the data were analyzed. Metabolites measured by positive and negative ion channels were combined, and the dimension reduction of the data was analyzed based on partial least squares discriminant analysis (PLS-DA). According to the results of metabolite comparison, all the differentiated metabolites were displayed in a volcano map. MetaboAnalyst (v5.0) was used for pathway enrichment analysis to screen metabolic pathways with significant changes 39.

### Statistical analysis

All data contained at least 3 independent replicates and the results are presented as mean ± standard error of the mean (SEM). Significant differences were determined by t-test or one-way analysis of variance (ANOVA) using GraphPad Prism 8 software (GraphPad Software Inc., San Diego, CA); values with *P < 0.05 and **P < 0.01 were considered to have significant and highly significant differences, respectively.

## Results

### Sleep deprivation caused a reduction of body weight and gut dysbiosis

**Figure 1:**
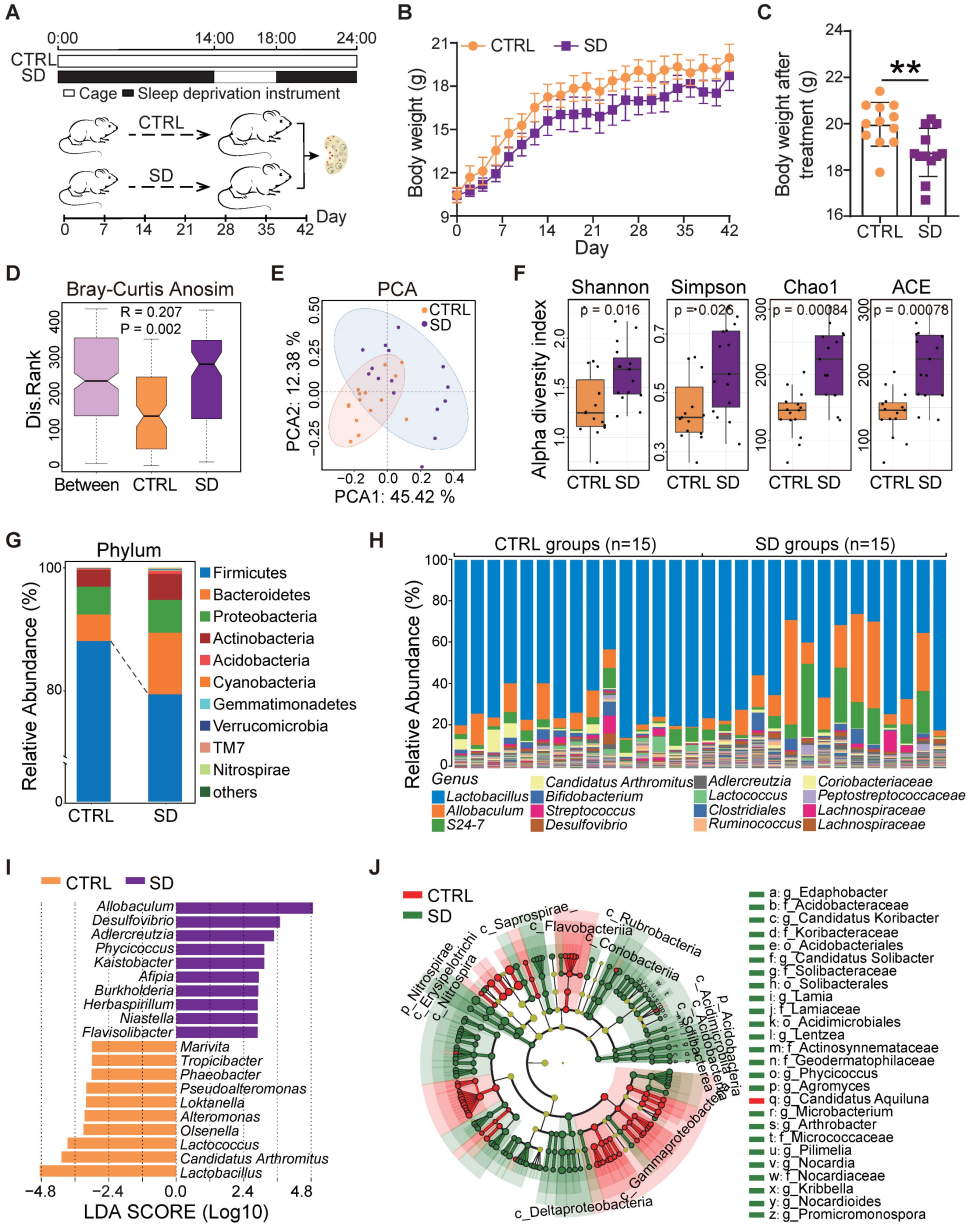
** Body weight and gut microbiota changes caused by sleep deprivation.** (A) Schematic diagram of the sleep deprivation (SD) experiment. (B) Changes of body weight in control (CTRL) and SD females during the experimental time (n=12). (C) Body weight of CTRL and SD females at the end of the experimental time (n=12). (D) ANOSIM similarity analysis of gut microbiota obtained from CTRL and SD females at the end of the experimental time. (E) PCA of the data shown in D. (F) α-Diversity indexes, including Shannon, Simpson, Chao1, and ACE of the data shown in D. (G) Relative abundance of the bacterial phyla in the gut microbiota of CTRL and SD females. (H) Relative abundance of bacterial genera in the gut microbiota of CTRL and SD females. (I) LDA algorithm of CTRL vs SD gut microbiota bacterial genera with an LDA score threshold of 2.0. (J) Cladogram of the main taxa of microbiota in CTRL and SD females based on LEfSe analysis.

SD was induced in female mice from 3 to 9 weeks of age, the period of adolescence critical for establishment of the ovarian reserve and its dynamic control 40, 41. During the 6-week experimental period, we registered a constant body weight increase and decrease in control (CTRL) and SD mice, respectively (Figure 1A-B). Accordingly, at the end of the treatment, the body weight of SD females was significantly lower than CTRL (Figure 1C).

The impact of SD on the gut microbiota was investigated by 16S rRNA sequencing of the small intestine contents obtained from CTRL and SD mice at the end of treatment. Analysis of similarities (ANOSIM) indicated that differences between CTRL and SD groups were significantly greater than differences within groups (Figure 1D). The PCA scatter plot confirmed significant changes within the gut microbiota caused by SD (Figure 1E). Moreover, the α-diversity analysis, including Shannon, Simpson, Chao1, and ACE indices, confirmed distinct differences in the abundance of microbial species between CTRL and SD groups (Figure 1F). Namely, bacterial species annotation data showed that SD altered the abundance of gut microbes both at the phylum and genus level (Figure 1G-H). Among phyla, *Firmicutes* and *Verrucomicrbia* decreased while *Bacteroides*, *Proteobacteria,* and *Actinobacteria* increased. To identify the specific microbiota related to SD, and using the linear discriminant analysis (LDA) effect size (LEfSe) method, we screened out 120 biomarkers. The top genera in SD were *Allobaculum, Desulfovibrio,* and* Adlercreuzia;* whereas in CTRL, *Lactobacillus*, *Candidatus Arthromitus,* and *Lactococcus* prevailed (Figure 1I-J). On this basis, we also employed STAMP software to conduct the difference analysis based on the abundance of gut microbes. In total, 102 types of differential bacterial genera were found, of which 64 genera were up-regulated after SD, including *Kaistobacter*, *Streptomyces*, *Flavisolibacter*, *Arthrobacter*, and *Promicromonospora*; meanwhile, 38 genera were down-regulated, including *Pseudoalteromonas*, *vibrio*, *Alteromonas*, *Octadecabacter*, and *Phaeobacter* (Figure S1A). Moreover, based on analysis of LEfSe and STAMP results, 96 bacteria genera were found in both groups (Figure S1B and Table S3). Among them, 12 types of the genera were selected and displayed, 6 of which were up-regulated and 6 down-regulated by SD (Figure S1C). These results indicated that there were substantial differences in the gut microbiome between SD and CTRL groups.

### Sleep deprivation impacted on intestinal immunity and the integrity of the intestinal epithelial barrier

**Figure 2:**
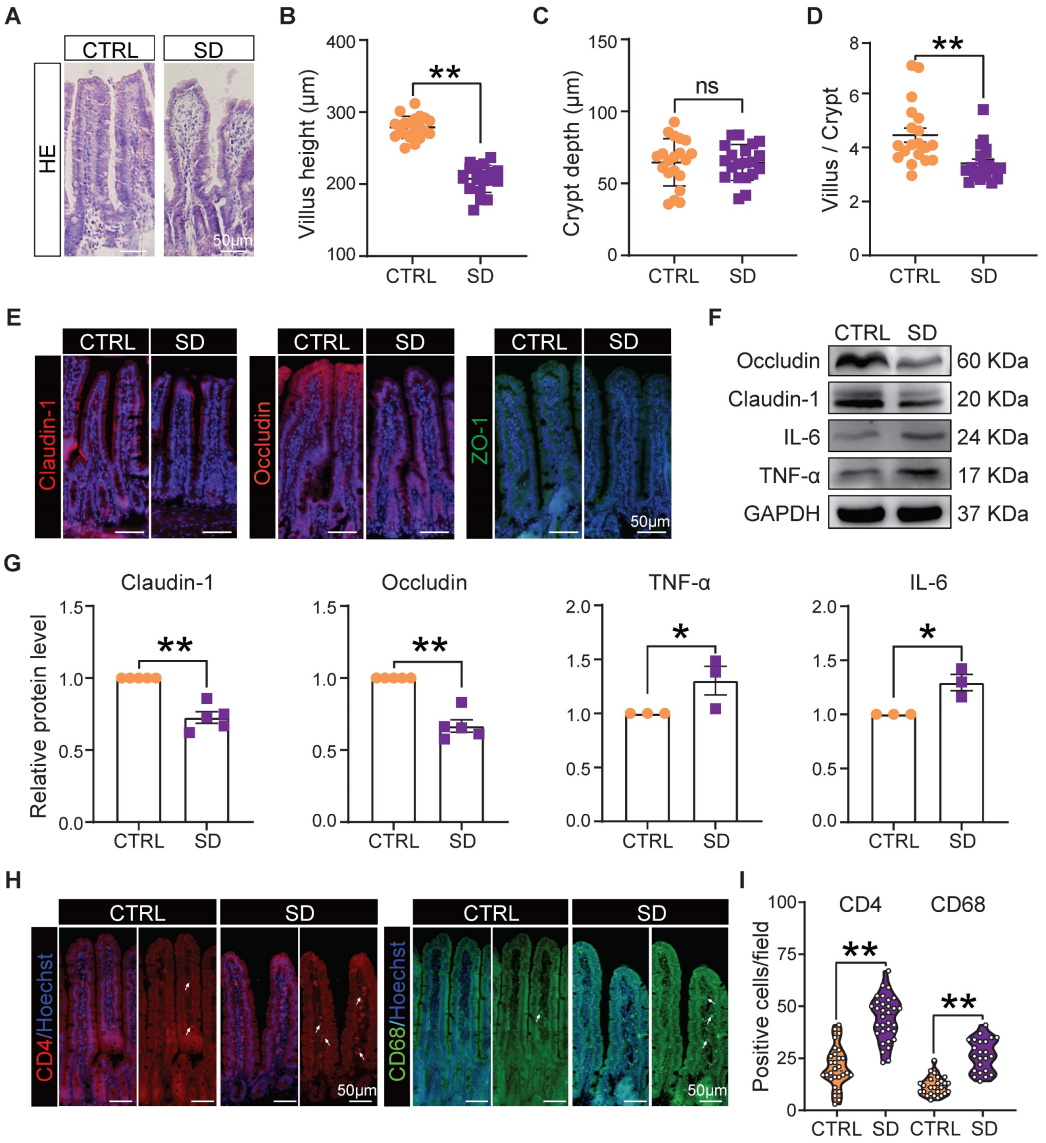
** Intestinal barrier disturbance caused by sleep deprivation.** (A) Representative H&E histological sections of small intestine samples collected from CTRL and SD females. (B-D) Comparison of villi height, crypt depth, and villi height/crypt depth of small intestine samples of CTRL and SD females. (E) Fluorescent immunostaining (IF) for the indicated tight junction proteins on small intestinal sections of CTRL and SD females. (F-G) Representative WB and relative densitometric evaluation of the amount of the indicated tight junction proteins and inflammatory cytokines in the small intestine of CTRL and SD females. (H-I) IF quantitative statistics of CD4 and CD68 proteins associated with intestinal immune cells.

It is well known that the integrity of the intestinal epithelial barrier is essential for maintaining a healthy symbiosis between the epithelial cells and the gut bacteria, and that tight junction proteins play a key role in maintaining barrier integrity. Thus, we collected histological samples of the small intestine and evaluated the expression of tight junction proteins in this organ of CTRL and SD mice. Histological sections showed that, after SD, the height of intestinal villi decreased significantly and, while the crypt depth did not change significantly, the ratio of villi height to crypt depth decreased significantly. Moreover, the epithelial layer appeared less compact, and cells showed hill-defined borders (Figure 2A-D). Most importantly, after SD, the fluorescence intensity of tight junction proteins such as Claudin-1, Occludin, and ZO-1 in tissue sections decreased, while their presence, as detected by WB, was reduced (Figure 2E-G). Interestingly, these changes were associated with increased levels of the inflammatory proteins interleukin-6 (IL-6) and tumor necrosis factor-alpha (TNF-α) (Figure 2F-G), a further indication of altered integrity of the epithelial barrier. In addition, an accumulation of CD4^+^ T cells and CD68^+^ macrophages was found in the lamina propria of the small intestine in sleep-deprived mice (Figure 2H-I), suggesting that SD increased immune cell infiltration in the lamina propria of the small intestine.

### Sleep deprivation caused systemic metabolic changes

**Figure 3:**
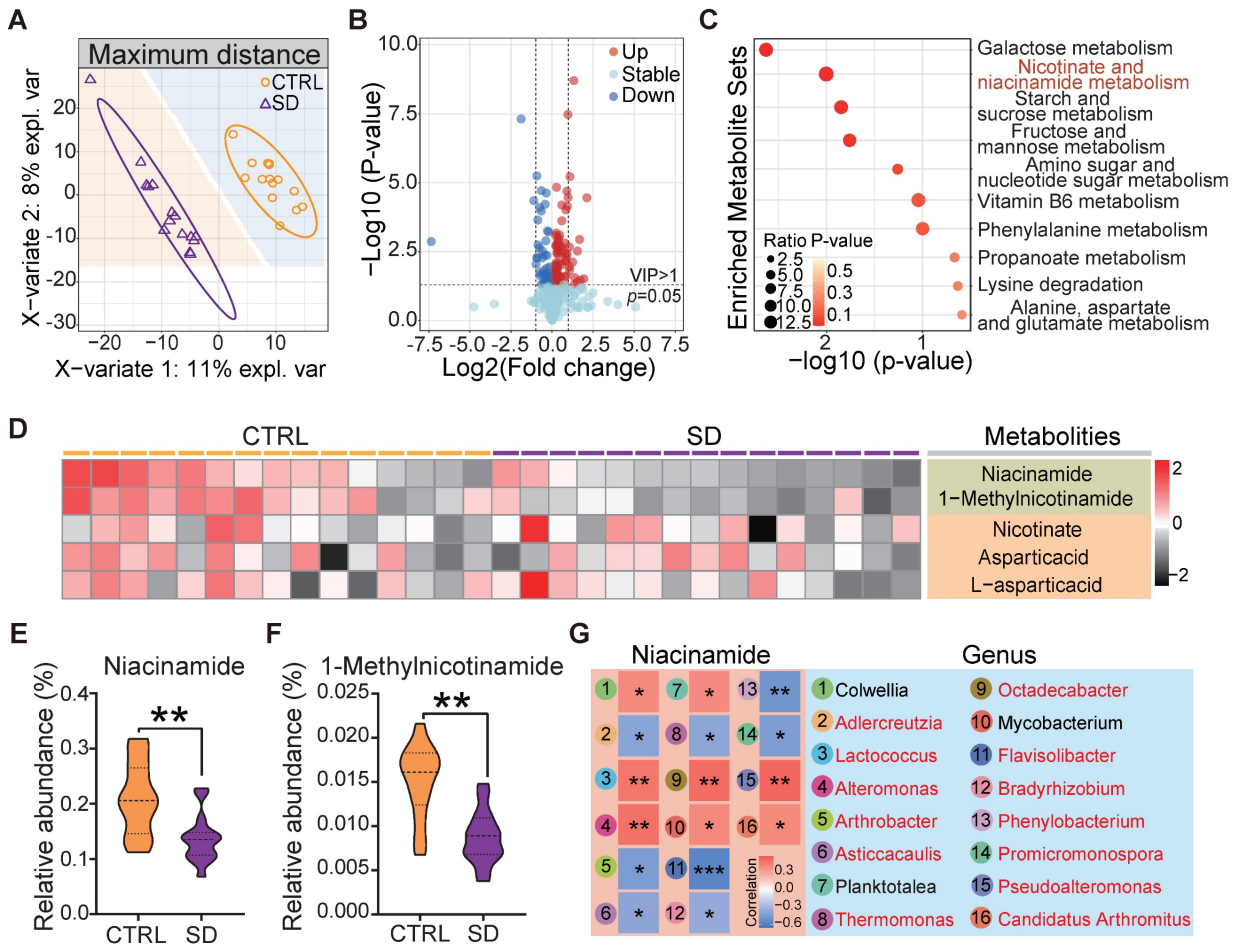
** Impact of sleep deprivation on the metabolome.** (A) PLS-DA discriminant analysis of the metabolome in blood serum from CTRL and SD females. (B) Volcano map of metabolite changes caused by SD. (C) KEGG metabolic pathway enrichment analysis. (D) Heatmap of metabolites in niacinamide (NAM) metabolic pathways. (E-F) Comparison of the relative abundance of NAM and 1-Methylnicotinamide in the metabolome of CTRL and SD females. (G) Spearman correlation analysis between NAM and different bacterial genera. The red font represents the bacteria genera with significant differences shown between CTRL and SD groups.

The gut microbiota plays a vital role in its host's metabolism 28. Several studies support the notion that metabolic processes are correlated with sleep and that SD can cause maladaptive metabolic changes 42. In order to verify how our SD model impacted on systemic metabolism, we examined the untargeted metabolome profile of blood serum samples obtained from CTRL and SD mice. Partial least squares discriminant analysis (PLS-DA) of the 1022 identified metabolites resulted in distinct metabolite clustering in CTRL and SD groups (Figure 3A). Using an orthogonal PLS-DA (OPLS-DA) model to obtain the variable importance for the projection (VIP) with OPLS-DA VIP >1 and P-value < 0.05, 122 differential metabolites were identified, 82 of which were up-regulated and 40 down-regulated after SD (Figure 3B). According to pathway annotation results of the Kyoto Encyclopedia of Genes and Genomes (KEGG) database, a total of 18 metabolic pathways were enriched in the differential metabolites between the 2 groups, including the metabolism of galactose, nicotinate and nicotinamiade/niacinamide (NAM), starch and sucrose, and fructose and mannose. Among them, nicotinate and NAM metabolic pathways had the highest enrichment rates (Figure 3C). The nicotinate and NAM pathways in the CTRL and SD groups are shown in Figure 3D. Namely, the relative abundance of serum NAM and 1-Methylnicotinamide was higher in CTRL than in the SD group (Figure 3E-F). To investigate the functional relationship between gut microbial dysbiosis and NAM metabolism, Spearman's correlation analysis was performed. As shown in Figure 3G, Spearman's correlation analysis between NAM and genus level bacteria showed that a total of 16 bacteria genera were significantly correlated with NAM, among which 13 bacteria genera were significantly different between CTRL and SD groups (Figure S1B).

### Sleep deprivation affected follicle number and dynamics and increased granulosa cell apoptosis

**Figure 4:**
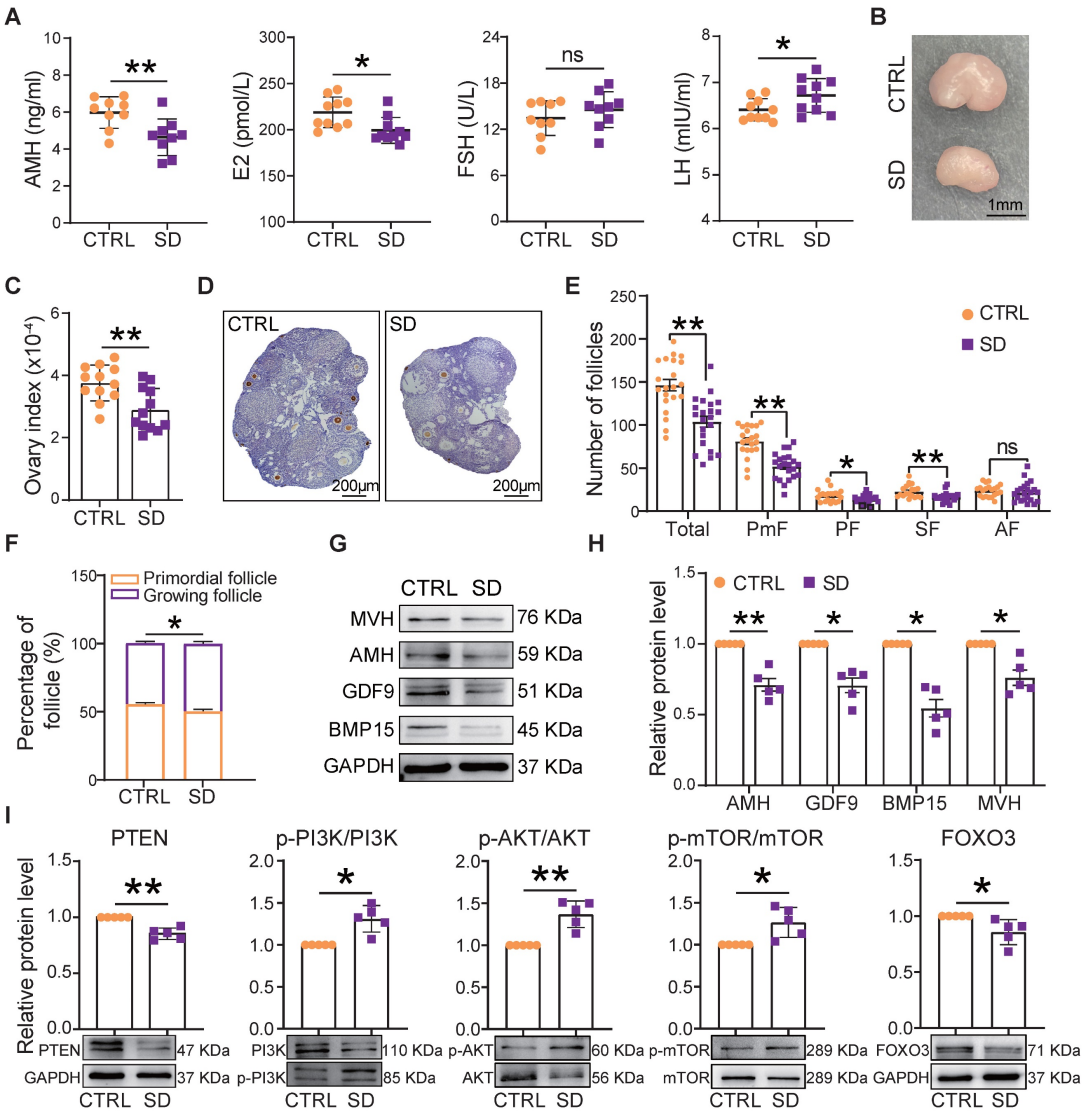
** Effects of sleep deprivation on ovary index and follicle dynamics.** (A) Blood serum concentrations of AMH, E2, FSH, and LH hormones at the end of the experimental time. (B) Representative picture of the CTRL and SD ovary at the end of the experimental time, bar =1 mm. (C) Ovary and body weight ratio (ovary index) in CTRL and SD females at the end of the experimental time (n=12). (D) Representative IHC for the oocyte-specific MVH protein on CTRL and SD ovary sections, bar = 200 μm. (E) Number of different classes of follicles in the 9 sections of CTRL and SD ovaries at the end of the experimental time. (F) Percent of primordial follicles and growing follicles (all other follicle classes, see below) for sections of CTRL and SD ovaries at the end of the experimental time. (G-I) Representative WB and relative densitometric evaluation of the amount of the indicated proteins from CTRL and SD ovaries at the end of the experimental time. PmF: Primordial follicle, PF: Primary follicle, SF: Secondary follicle, AF: Antral follicle.

At the end of treatment of SD females, the blood concentration of AMH and E2 decreased, while that of LH increased compared to CTRL (Figure 4A). Moreover, in parallel to the decrease in body weight, we found a significant reduction in ovary size and index (ovary and body weight ratio) in comparison to CTRL (Figure 4B-C). The SD effects on the ovary were further investigated by comparing the number of follicles and the percent of each follicle class in CTRL and SD females (Figure 4D and Figure S2A).

Compared with CTRL, after only 4 weeks of SD treatment, the number of follicles in the 9 scored sections of SD ovaries had decreased significantly, mostly through a marked reduction in the number of PmF, representing the ovarian reserve (Figure S2B). At 6 weeks, this trend was even more pronounced (Figure 4E), and the percent of growing follicles appeared slightly higher in SD (49.66%±1.50%) than CTRL (44.92%±1.19%) ovaries (Figure 4F), suggesting an increment in PmF activation. In line with such a possibility, serum levels of AMH (a marker of ovarian reserve) in SD females were significantly lower than in CTRL (Figure 4A). Moreover, both for mRNA and proteins, the expression of AMH as well that of oocyte specific proteins [growth differentiation factor 9 (GDF9), bone morphogenetic protein 15 (BMP15), and mouse vasa homolog (MVH)] were reduced in SD compared with CTRL ovaries (Figure 4G-H and Figure S2C). At the same time, markers of PmF activation including phosphorylated PI3K, AKT, and mTOR proteins were increased whereas the levels of critical inhibitors of PmF activation including PTEN and FOXO3 proteins, decreased (Figure 4I).

In SD ovaries, besides indication of PmF activation, we found that the number of TUNEL-positive cells, mainly among granulosa cells, were significantly increase in comparison to CTRL (Figure S3A-B). Raised amounts of γ-H2AX protein, a marker of DNA damage, and proapoptotic markers such as BAX/BCL2, P53 and cleaved Caspase 9 paralleled this increase (Figure S3C-D).

### Sleep deprivation impaired oocyte mitochondrial function and meiotic maturation

**Figure 5:**
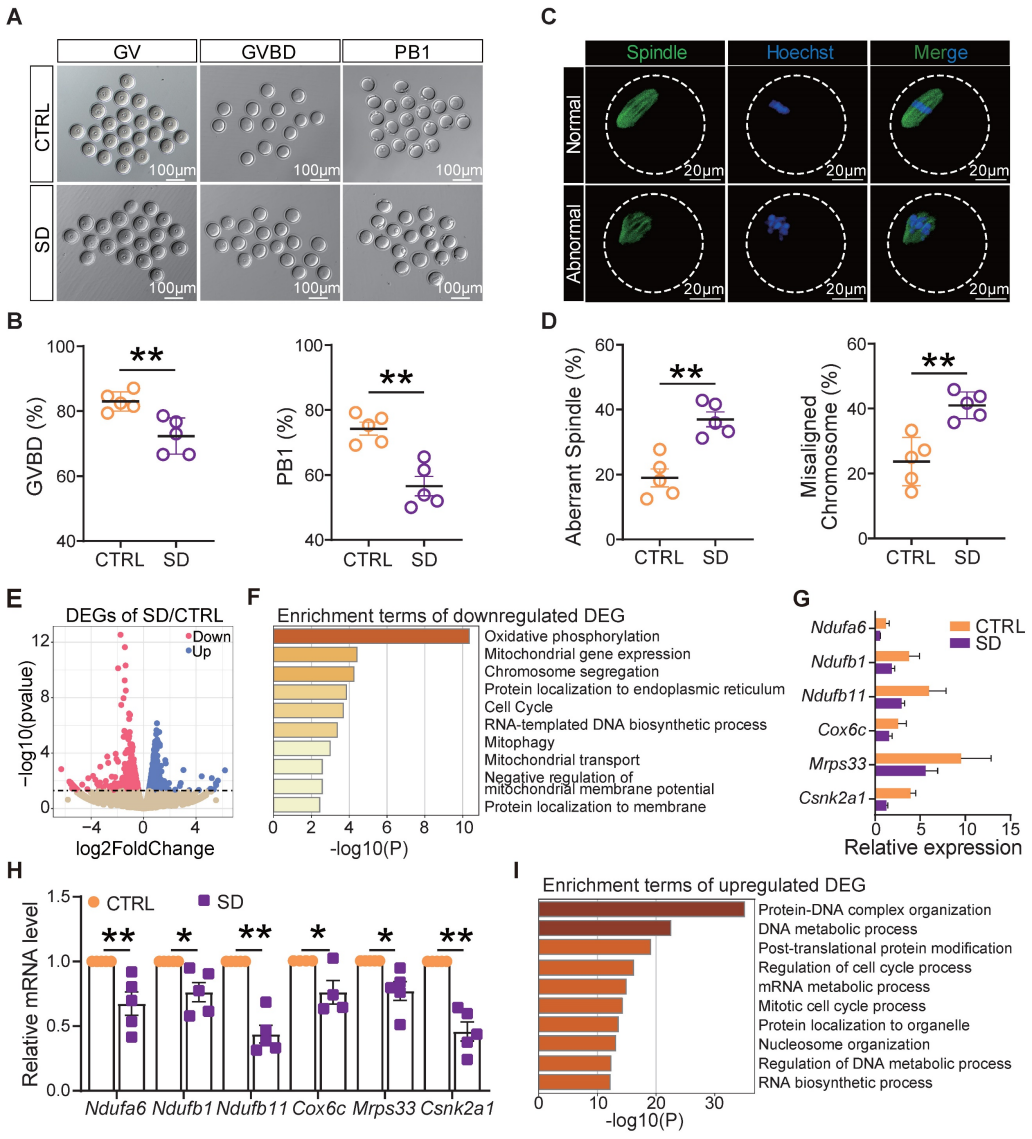
** Sleep deprivation affected oocyte maturation, spindle assembly, and mitochondrial function.** (A) Representative pictures of GV, GVBD, and PB1. Scale bar = 100 μm. (B) Percent of GVBD and PB1 in CTRL and SD oocytes after *in vitro* culture for 4-6 h and 12-16 h (n>90). (C) Representative pictures of normal and abnormal spindle morphologies and chromosome alignment. (D) Percent of aberrant spindles and misaligned chromosomes in CTRL and SD oocytes after *in vitro* culture for 8 h (n>90). (E) Volcano map of gene transcript differences in CTRL and SD oocytes. (F) Enrichment terms of down-regulated genes. (G) Gene expression levels of the indicated genes related to mitochondrial function. (H) RT-qPCR of the same genes showed in G. (I) Enrichment terms of up-regulated genes.

To measure the influence of SD on oocyte quality, we compared the ability of fully grown germinal vesicle (GV) oocytes cultured* in vitro* from CTRL and SD ovaries to resume meiosis [as germinal vesicle break-down (GVBD)], reach metaphase II (extrusion of PB1), and to correctly assemble the meiotic spindle. The results reported in Figures 5A and 5B show that a smaller number of SD oocytes underwent GVBD and extruded PB1 in comparison to CTRL. Moreover, the percent of spindle (Metaphase I) abnormalities and chromosome misalignments increased significantly in SD oocytes after 8 h of culture (Figure 5C-D).

In order to identify molecular pathways or altered functions, we next performed single-cell transcriptome sequencing (scRNA-seq) analyses of CTRL and SD oocytes. Among 1640 differentially expressed genes, we found 1006 genes up-regulated and 634 genes down-regulated in SD oocytes (Figure 5E). Interestingly, down-regulated genes were mainly enriched in terms of oxidative phosphorylation, mitochondrial gene expression, chromosome segregation, and mitophagy signaling pathways (Figure 5F). Deleterious effects of SD on the mitochondrial function of oocytes were supported by reductions in mRNA levels of *Ndufa6*,* Ndufb1*,* Ndufb11*, *Cox6c*, *Mrps33*, and *Csnk2a1* involved in oxidative phosphorylation and respiratory chain complex assembly, mitochondrial translation, and mitophagy (Figure 5G-H). Up-regulated genes were mainly enriched in terms of protein-DNA complex organization, DNA metabolic process, and regulation of cell cycle process pathways (Figure 5I).

In addition, using RNA-seq analysis of the whole ovaries and c-means clustering algorithm, 8 gene modules were generated (Figure S4A). Notably, most of the genes in Cluster 1, mainly enriched in mitochondrial function, appeared downregulated in SD ovaries (Figure S4B), which is consistent with scRNA-seq results of oocytes, while several genes in cluster 7 were mainly enriched in histone modification, and mRNA processing was up-regulated in SD ovaries (Figure S4C). We also identified 133 common differential genes both in RNA-seq and scRNA-seq (Figure S4D), which are involved in the regulation of growth and development, chromosome organization, and ovarian infertility (Figure S4E). In addition, the expression pattern of POI-related genes in the ovaries also changed significantly after SD (Figure S4F).

### Fecal microbiota transplantation in host females recapitulated ovarian damage induced by sleep deprivation

**Figure 6:**
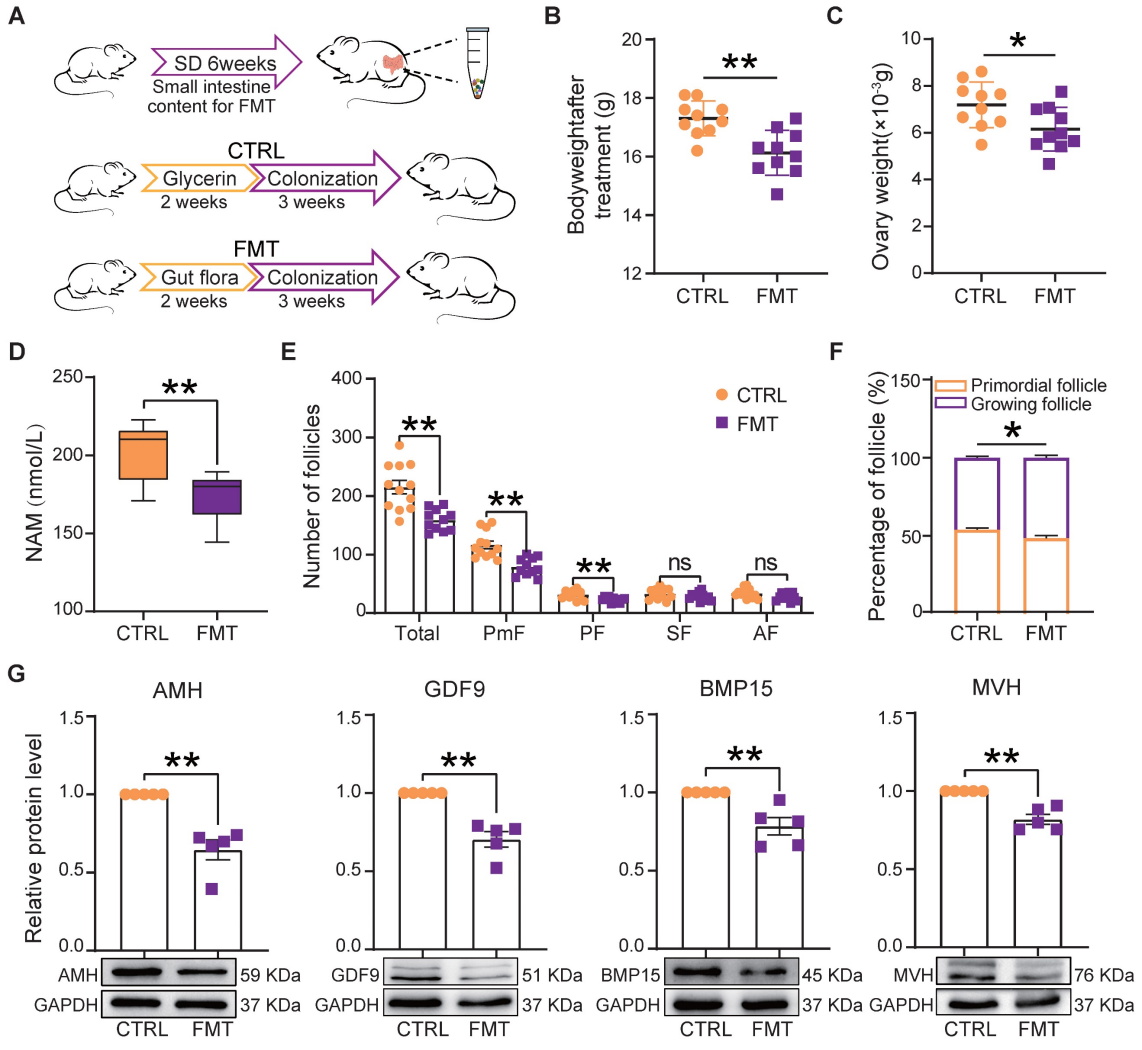
** Fecal microbiota transplantation from SD females reproduces the effects of SD in normal host females.** (A) Study design of fecal microbiota transplantation** (**FMT). (B-C) Body and ovary weights of females under the indicated experimental conditions. (D) Concentration of niacinamide (NAM) in the blood serum obtained from females under the indicated experimental conditions. (E) Total number and different follicle class number for sections of ovaries from females under the indicated experimental conditions. (F) Percent of PmF and growing follicles in the ovaries of females under the indicated experimental conditions. (G) Representative WB and relative densitometric evaluation of the amount of the indicated proteins in the ovaries of females under the indicated experimental conditions.

Fecal microbiota transplantation (FMT) is used to directly change the recipient's gut microbiota 31, 43. In order to understand whether changes of gut microbiota caused by SD could be responsible for the ovary disorders described in the previous sections, we transplanted the fecal microbiota of SD females to normal mice by oral gavage (Figure 6A). After 2 weeks of feeding and 3 weeks of colonization, we found that in FMT females blood serum concentrations of NAM were significantly lower than in CTRL (Figure 6D). Furthermore, the body and ovary weights of recipient females were lower than those of CTRL and comparable to those of SD mice reported in Figure 1C and Figure 4C (Figure 6B-C). Notably, the ovary indices of CTRL and FMT females were not significantly different (not shown).

Moreover, FMT females showed follicle alterations similar to those induced by SD including reduced number (mainly PmF), and altered dynamics (Figure 6E-F), associated with decreased expression of AMH, GDF9, BMP15, and MVH proteins (Figure 6G).

### Supplementation of niacinamide alleviated the effects of sleep deprivation on follicle dynamics and female reproductive performance

**Figure 7:**
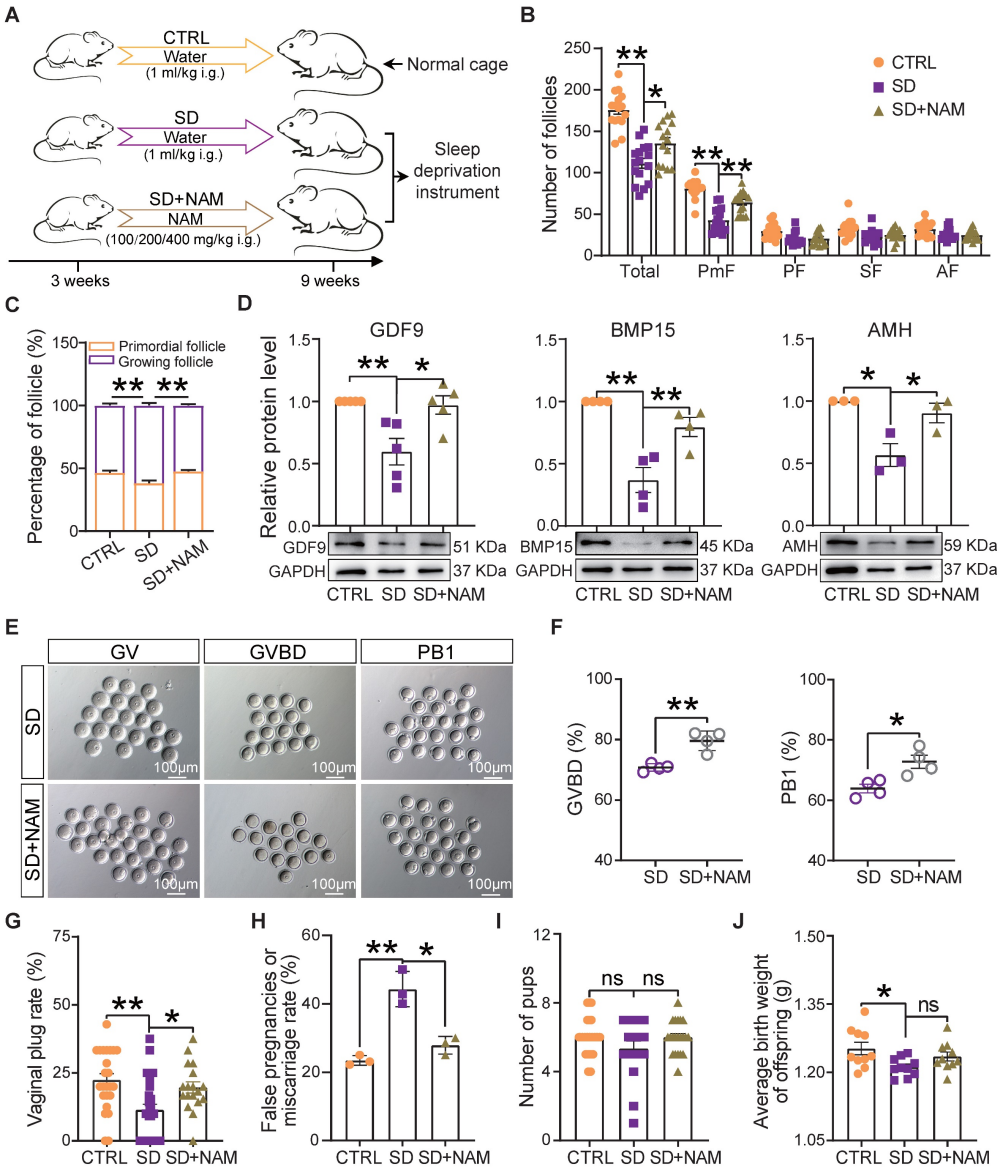
** Dietary niacinamide supplementation alleviated the effects of sleep deprivation on the ovarian follicle reserve.** (A) Study design of niacinamide (NAM) supplementation. (B) Total number and different follicle class number for sections of ovaries of females under the indicated experimental conditions. (C) Percent of PmF and growing follicles in the ovaries of females under the indicated experimental conditions. (D) Representative WB and relative densitometric evaluation of the amounts of the indicated proteins in the ovaries of females under the indicated experimental conditions. (E) Representative pictures of oocytes at GV, GVBD, and PB1 stages in SD and SD+NAM groups. Scale bar = 100 μm. (F) Percentage of oocytes at GVBD and PB1 stages in SD, and SD+NAM groups after *in vitro* culture (n>90). (G) The statistical effect of vaginal plug rate in CTRL, SD, and SD+NAM groups, also including false pregnancies or miscarriage rate (H, number of female mice with vaginal plugs in each group >29), pup numbers (I), and average birth weight of offspring (J).

It is well known that NAM, a nicotinamide adenine dinucleotide (NAD^+^) precursor, is an essential redox cofactor for cellular reactions in mammals and microbes. This is mostly due to the fact that circulating NAM enters the gut lumen and is able to support microbial NAD^+^ synthesis 44. For this reason and since we found NAM deficiency to be attributable to a specific gut microbiota imbalance, as one of the most evident effects of our SD treatment in the female mice, we performed experiments to investigate whether NAM supplementation could counteract the effects of SD on follicle dynamics described in the previous sections (Figure 7A). The results showed that although the feeding of 100-400 mg/kg NAM for 6 weeks had no significant effect on body and ovary weight of mice (Figure S5A), the ovaries of SD females fed with 200 mg/kg NAM had follicle numbers and percent of PmFs and of the other follicle classes comparable to those of CTRL (Figure 7B-C and Figure S5B-D). In these ovaries, the levels of AMH and the oocyte secreted factors GDF9 and BMP15 were at concentrations comparable to those of CTRL (Figure 7D). In addition, a higher proportion of oocytes from female mice supplemented with NAM reached GVBD and expelled PB1 compared to SD (Figure 7E-F). In terms of reproductive performance, we found a significant reduction in the rate of pregnant (marked by the presence of vaginal plug) and a significant increase in the rate of false pregnancy or miscarriage in SD female mice, which were effectively improved after NAM supplementation, although the indexes of litter size and average birth weight showed no significant difference (Figure 7G-J).

## Discussion

SD is a stressor that affects multiple tissues throughout the body. In recent years, research has shown that abnormal sleep patterns and duration affect systemic metabolism and gut microbiota composition. This latter in turn provides substrates for supplying and regulating multiple compounds reaching the circulation and influencing the function of distal organs. In the present study, using a validated device and female mice, we investigated the effects of SD on murine ovaries during the adolescence period when follicles, the ovarian reserve, and the basic dynamics and control of folliculogenesis are established.

We first confirmed that SD caused gut dysbiosis and systemic metabolic changes in the treated females. In line with other studies reporting mass death of intestinal epithelial cells, reduction of goblet cells, decreased expression of tight junction proteins, and impaired intestinal barrier function after SD 45-47, we obtained histological evidence suggesting the presence of an altered intestinal immunity and integrity of the intestinal epithelial barrier. Moreover, after performing 16S rRNA sequencing, relevant changes in the microbiota were detected. Our data showed that SD caused relevant changes in the abundance of gut microbes at the phylum and genus level. For example, following SD, bacteria genera including *Lactobacillus*,* Lactococcus*, *Candidatus arthromitus*, and *Pseudoalteromonas* decreased whereas* Allobaculum*, *Kaistobacter*,* Desulfovibrio*, and* Adlercreutzia* increased. As an important component of the digestive and female reproductive microbiota, *Lactobacillus* maintains microbiota balance, improves digestive function, assists the synthesis of B vitamins and vitamin K, inhibits pro-inflammatory mediators, and produces antibacterial activity against pathogens 48. *Candidatus arthromitus* is a commensal bacterium necessary for inducing the postnatal maturation of homeostatic innate and adaptive immune responses in the mouse gut 49. Interestingly, an increase in the relative abundance of *Allobaculum* after SD has also been observed in the intestines of mice fed a high-fat diet while an increase in *Desulfovibrio* is indicative of intestinal inflammation and altered sensitivity 50. Furthermore, *Desulfovibrio* is increased in a rotenone-induced Parkinson's mouse model and in a high-cholesterol-induced non-alcoholic fatty liver mouse model 51, 52.

SD females showed a significant body weight reduction and 18 metabolic pathways were enriched in differential metabolites in the blood of CTRL and SD animals. Among them, nicotinate and NAM pathways had the highest enrichment rates. The higher amounts of NAM present in CTRL in comparison to SD, were correlated to decreased numbers of *Lactococcus*, *Alteromonas*, *Pseudoalteromonas*, *Candidatus arthromitus*, and *Octadecabacter*, and an increase in *Arthrobacter*, *Adlercreutzia*, *Flavisolibacter*, *Phenylobacterium*, and *Promicromonospora* (Figure 3G). The decreased NAM content after FMT also confirmed the association between NAM and gut microbiome changes (Figure 6D). In this regard, the *Firmicutes*/*Bacteroidetes* (F/B) ratio is widely believed to play an important role in maintaining normal intestinal homeostasis 53-55. At the phylum level, higher *Bacteroides* and lower *Firmicutes* abundances were observed in POI patients 22, which coincided with the sequencing data after SD. In addition, studies on mice with chronic colitis found that NAM could increase the proportion of beneficial bacteria and restore the function of gut microbiota 56. Recent results indicate that intestinal bacteria can promote host NAD^+^ pools through deamidation and de novo synthesis pathways 57, 58. Our investigation, however, did not allow us to distinguish between these possibilities.

During the female reproductive cycle, only a limited number of primordial follicles are recruited, therefore disruption of primordial follicle activation inevitably leads to abnormal follicle development. When primordial follicles are activated too quickly, the size of the ovarian reserve is impacted 18. In general, POI is often induced by abnormal follicular oocyte development and ovarian dysfunction caused by premature depletion of follicle reserve and increased follicle atresia. Histological observations showed that SD reduced the follicle reserve, likely by increasing both primordial follicle activation and follicle atresia. Therefore, we suggest that SD induced the POI phenotype in adolescent female mice; these findings are also supported by the abnormal genetic changes associated with POI. At the same time, decreased ovarian size and changes in the levels of circulating sex hormones were observed. Similar to our findings, some studies suggest that a lack of sleep contributes to obesity and insulin resistance, which in turn promotes PCOS 59, 60. Furthermore, SD may cause harmful alterations to endocrine systems (including the ovarian axis) 61. In menstruating women, short sleep duration is associated with lower estradiol and luteal phase progesterone levels and an increased incidence of anovulation, which can also decrease reproductive capacity by impairing the arrival of morulas in the uterus 62.

In our study, the ability of fully grown oocytes to resume and complete meiosis I was also observed to be impaired. scRNA-seq analyses performed on SD oocytes indicated that mitochondrial disfunction was one cause of the reduced quality of such oocytes. This likely impacted on the reproductive performance of the SD females and was significantly reduced, as shown by the lower number of vaginal plugs and higher levels of false pregnancies or miscarriage.

The central role of microbiota impacting on systemic NAM levels and inducing disordered ovarian function, following the SD reported here, was clearly supported by FMT. In female hosts, FMT resulted in a marked reduction in NAM concentration in the blood, and ovarian disorders-including weight reduction, reduced follicle number (mainly PmF), and altered folliculogenesis dynamics-associated with the decrease in AMH, GDF9, BMP15, and MVH proteins.

Finally, the ability of NAM supplementation to counteract the effects of SD on follicle numbers and dynamics and the decrease of AMH, GDF9, and BMP15, points to the reduction of this vitamin as one of the main causes of ovarian disfunction resulting from SD. As reported above, NAM, as a precursor of NAD^+^, is a key component of the NAD^+^ salvage pathway and an essential redox cofactor for cellular reactions in mammals and microbes. Challapa *et al.*, using isotope labeling to track NAD metabolism in host tissues and gut microbiota, found that circulating host NAM enters the gut lumen and supports microbial NAD synthesis; then the microbiota subsequently converts host-derived NAM into nicotinic acid, which is used for NAD synthesis in host tissue 63. Relevant to the present results, continuous administration of NAM has been shown to maintain DNA integrity and prevent apoptosis 64. NAM treatment can increase the expression of proteins associated with mitochondrial function, including oxidative phosphorylation, fatty acid oxidation, and TCA cycling 65. Studies in patients with poor ovarian response (POR) have shown that this disfunction is related to nicotinate and NAM metabolic pathways and appears to impact the ovarian reserve 66. Moreover, NAM is very important for oocyte maturation, it is involved in the salvage pathway of NAD^+^ synthesis in cumulus cells, and is increased substantially throughout the course of oocyte maturation 67. We speculate that the mitochondrial disfunction observed in oocytes can be due, at least in part, to a decrease in NAM availability. Increasing NAD^+^ levels by supplementing NAD^+^ preforms, such as NAM, niacinamide ribonucleoside (NR), or niacinamide mononucleotide (NMN), may enhance mitochondrial function and promote nuclear and cytoplasmic maturation to mitigate fertility damage associated with ovarian aging 68. Interestingly, some studies show that the level of NAM in preovulatory follicles can be significantly higher than that in small follicles and that NAM concentration in the follicular fluid of antral follicles is positively correlated with oocyte maturation and fertilization rate. These findings support the notion that NAM may favor follicular development, oocyte quality, and reproductive performance by increasing endogenous NAD^+^ levels to improve mitochondrial function 69. In line with the present results, NAM supplementation improved oocyte quality and offspring development by modulating mitochondrial function in an aged *Caenorhabditis elegans* model 70.

## Conclusions

In conclusion, our results support the notion that, in adolescent females, SD can cause changes in the gut microbiota that impact on systemic metabolism centered on NAM pathways. In the ovary, this can impair oocyte mitochondrial energy pathways resulting in POI and reduced oocyte meiotic maturation. Finally, NAM supplementation can be used to counteract the deleterious effects of SD on the ovary.

## Supplementary Material

Supplementary figures and tables.

## Abbreviations

POI: premature ovarian insufficiency
PmF: primordial follicles
SD: sleep deprivation
FMT: fecal microbiota transplantation
NAM: nicotinamiade/niacinamide
NAD^+^: nicotinamide adenine dinucleotide
GV: germinal vesicle
GVBD: germinal vesicle break-down
PBE: polar body extrusion
ANOSIM: analysis of similarities
IHC: immunochemistry
RT-qPCR: real-time quantitative PCR
OTUs: operation altaxonomic units
PCoA: principal coordinate analysis
LEfSe: linear discriminate analysis effect size
VIP: variable importance for the projection
PmF: primordial follicle
PF: primary follicle
SF: secondary follicle
AF: antral follicle
LH: luteinizing hormone
