# Supplementary material for: Sleep deprivation causes gut dysbiosis impacting on systemic metabolomics leading to premature ovarian insufficiency in adolescent mice
Source: Theranostics. 2024 Jun 17;14(9):3760–76. doi: 10.7150/thno.95197 (PMC11209713; doi:10.7150/thno.95197)
Supplement: Supplementary file 1 — Supplementary figures and tables. [file thnov14p3760s1.pdf]

Supplementary Figures

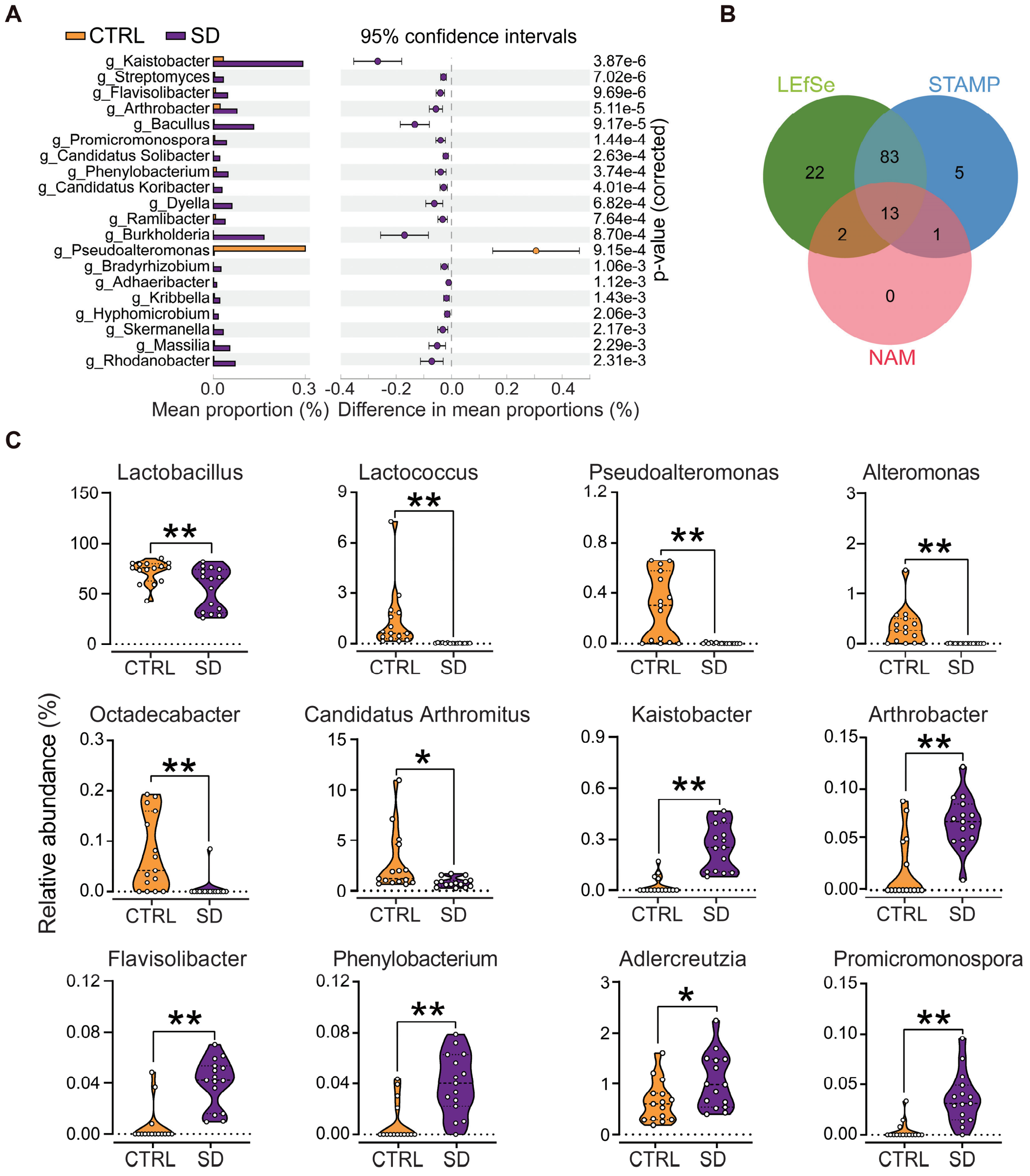

Figure S1. Changes in the microbiome after sleep deprivation

(A) A total of 102 different bacteria genera were selected by STAMP (Top20). (B) The intersection of bacteria selected by LEfSe and STAMP, and the intersection with NAM-related bacteria. (C) Bacteria genera that were significantly up-regulated or down-regulated after SD.

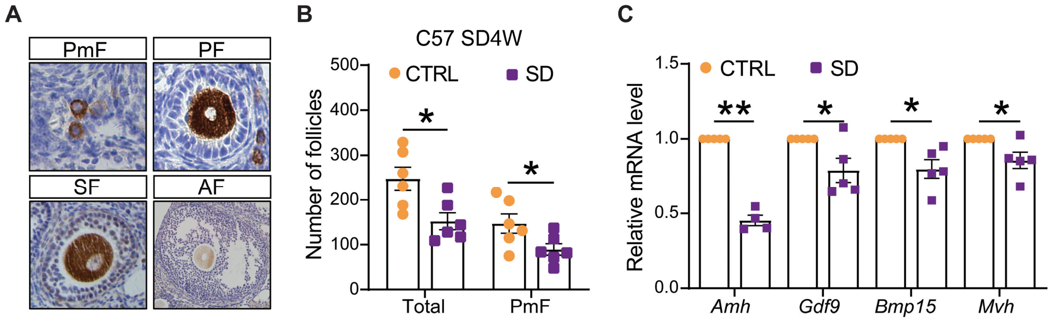

Figure S2. Effects of SD on the number of ovarian follicles

(A) Representative images of primordial follicle (PmF), primary follicle (PF), secondary follicle (SF), and antral follicle (AF). (B) Total follicle and PmF number for sections of ovaries of females after SD for 4 weeks. (C) Levels of *Amh*, *Gdf9*, *Bmp15,* and *Mvh* mRNAs in CTRL and SD ovaries at the end of the experimental time.

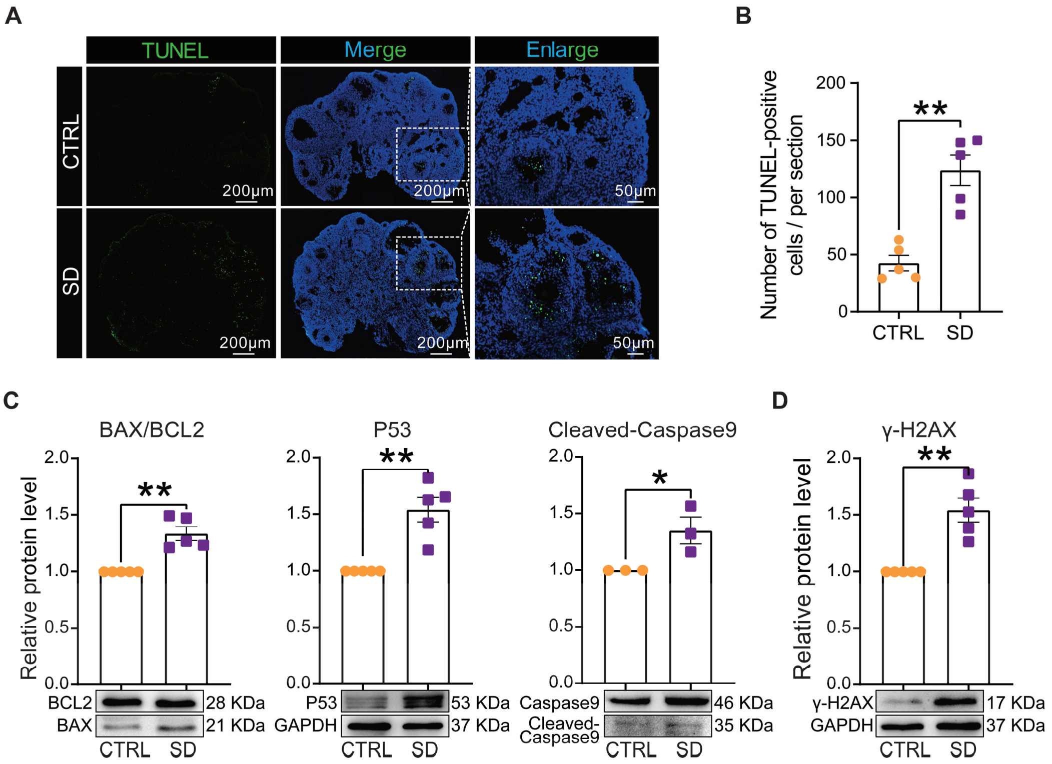

Figure S3. Increased apoptotic markers and DNA damage in the ovaries of SD females

(A) Representative sections of CTRL and SD ovaries after TUNEL (green) and Hoechst (blue) nuclei staining. Scale bar = 200 and 50 μm. (B) Total number of TUNEL-positive cells/section in CTRL and SD ovaries. (C-D) Representative WB and relative densitometric evaluation of the amount of the indicated proteins in CTRL and SD ovaries.

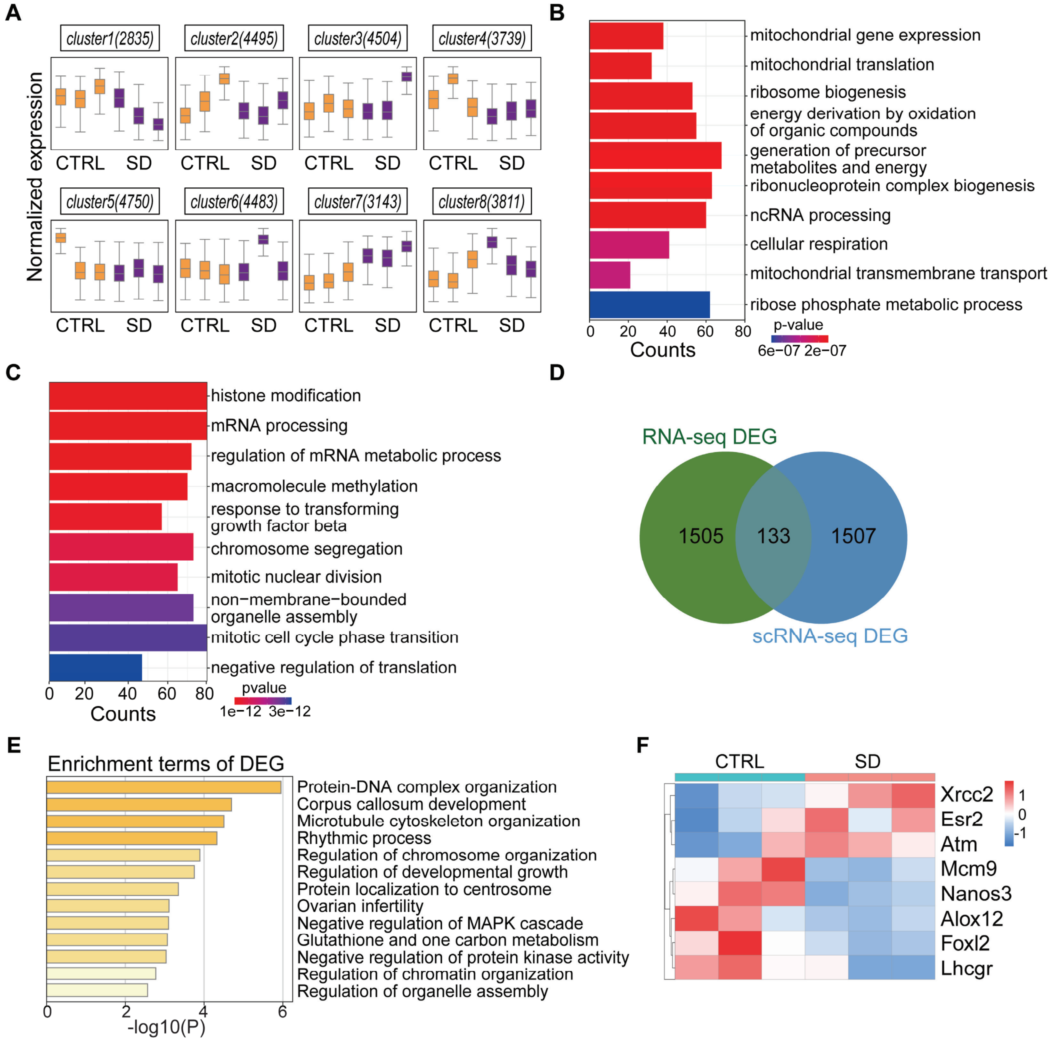

Figure S4. Comparison of RNA-seq data obtained from CTRL and SD ovaries

(A) Cluster analysis of the gene expression trend from RNA-seq data of the ovary. (B) KEGG enrichment result of genes in cluster 1. (C) KEGG enrichment result of genes in cluster 7. (D) Intersection of differential genes in bulk RNA-seq and scRNA-seq. (E) Pathway enrichment results of differential genes contained in both RNA-seq and scRNA-seq. (F) Changes in POI-related pathogenic genes in the ovary.

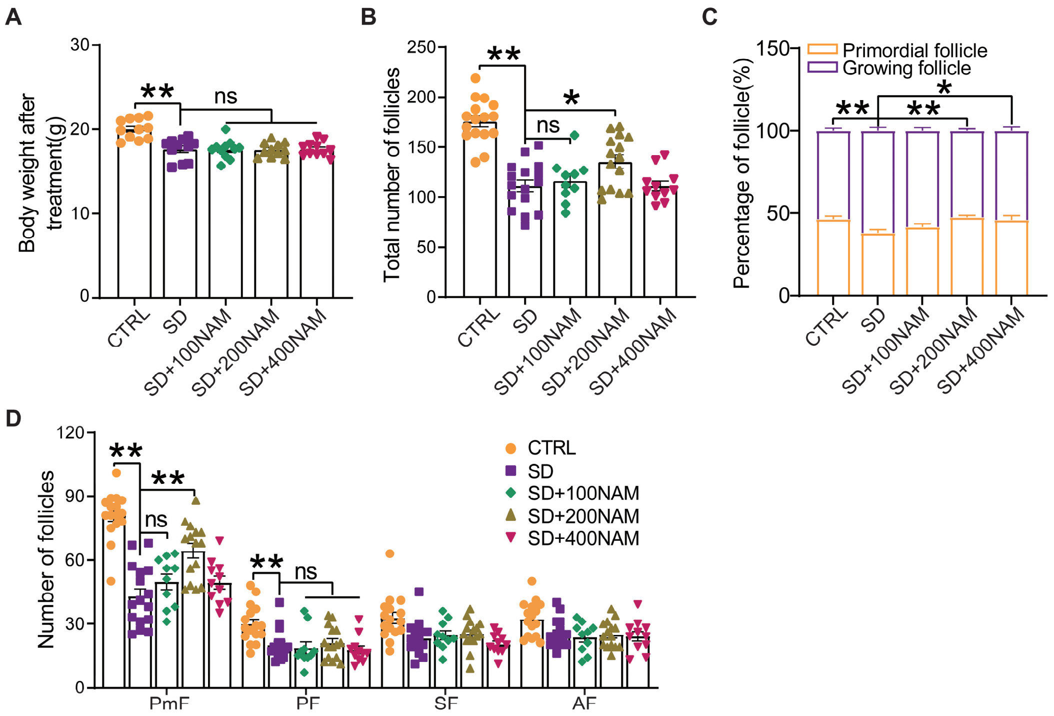

Figure S5. Concentration dependence of the anti-SD effects of niacinamide supplementation

Different recoveries of body weight (A), total follicle number (B), ratio of PmF/growing follicles, and number of different follicle classes (C-D) to CTRL values following 3 levels of niacinamide (NAM) supplementation to SD females.

Table S1. Antibody information used in the study.

Primary antibodies	Manufacturer and Product code	Dilution	Source
GDF9	Abcam (ab93892)	WB 1:1000	Rabbit
BMP15	Abcam (ab108413)	WB 1:1000	Rabbit
P53	Abcam (ab26)	WB 1:500	Mouse
Caspase9	Abcam (ab202068)	WB 1:1000	Rabbit
γ-H2AX	Abcam (ab26350)	WB 1:1000	Mouse
MVH	Abcam (ab13840)	WB 1:1000 IHC 1:200	Rabbit
AMH	ABclonal (A8538)	WB 1:1000	Rabbit
PI3K	ABclonal (A0265)	WB 1:1000	Rabbit
ZO-1	ABclonal (A0569)	WB 1:1000 IF 1:200	Rabbit
Occludin	ABclonal (A2601)	WB 1:1000 IF 1:200	Rabbit
Claudin-1	ABclonal (A21971)	WB 1:1000 IF 1:200	Rabbit
CD4	ABclonal (A23259)	IF 1:200	Rabbit
p-PI3K	ABclonal (A11177)	WB 1:1000	Rabbit
GAPDH	Affinity (AF7021)	WB 1:2000	Rabbit
p-AKT	Affinity (AF0016)	WB 1:1000	Rabbit
BCL2	Beyotime (AB112)	WB 1:1000	Rabbit
FOXO3	Novusbio (NBP2-16521)	WB 1:1000	Rabbit
AKT	Sangon Biotech (D155317)	WB 1:500	Rabbit
PTEN	Sangon Biotech (D261095)	WB 1:500	Rabbit
IL-6	Affinity (DF6087)	WB 1:1000	Rabbit
CD68	Affinity (DF7518)	IF 1:200	Rabbit
TNF-α	Affinity (AF7014)	WB 1:500	Rabbit
BAX	Cell Signaling Technology (2772S)	WB 1:1000	Rabbit
mTOR	Cell Signaling Technology (2983)	WB 1:1000	Rabbit
p-mTOR	Cell Signaling Technology (5536)	WB 1:1000	Rabbit

Secondary antibodies	Manufacturer and Product code	Dilution	Source
FITC-conjugated goat anti-rabbit (IF)	Beyotime (A0562)	1:200	Goat
HRP-conjugated goat anti- rabbit IgG (WB)	Beyotime (A0208)	1:1000	Goat
HRP-conjugated goat anti- Mouse IgG (WB)	Beyotime (A0216)	1:1000	Goat
Goat anti-mouse IgG H&L (Alexa Fluor®488)	Abcam (ab150113)	1:200	Goat
Goat anti-rabbit IgG H&L (Alexa Fluor®555)	Abcam (ab150078)	1:200	Goat

Table S2. Primers used for quantitative RT-PCR.

Gapdh-F	GTCATTGAGAGCAATGCCAG
Gapdh-R	GTGTTGCTACCCCCAATGTG
Amh-F	CTCATCCCGGAGACCTACCA
Amh-R	GCGAGCCTGCATTTTTAGCA
Gdf9-F	TCTTAGTAGCCTTAGCTCTCAGG
Gdf9-R	TGTCAGTCCCA TCTACAGGCA
Bmp15-F	TCCTTGCTGACGACCCTACA T
Bmp15-R	TGAGGCTTAAGTGGTCTTGCA
Mvh-F	TCAGACGCTCAACAGGATGT
Mvh-R	ACTGGA TTGGGAGCTTGTGA
Ndufb1-F	CCAGGCTGAAGCAGTCAAGA
Ndufb1-R	GACAAATCCCGCAGGGACAA
Ndufa6-F	AGTATGGAAGCAGCGGACAC
Ndufa6-R	ATGCACCTTCCCATCAGGTG
Ndufb11-F	AAATAGAGTCCGCACCTCGC
Ndufb11-R	GTACTGCGGAGTCCTTGCTA
Cox6c-F	GGTCCTCCATCGACTCTTGC
Cox6c-R	CCAGAAGACCACGCATCTGT
Mrps33-F	TCTCCGCTTTCGGAGTATGC
Mrps33-R	TGCTCATCTCACTTTCATGGACT
Csnk2a1-F	CAGGTACCGTGGGAACCG
Csnk2a1-R	GTGTAAACTCTGGCCCTGCT

Table S3. Genus of bacteria in LEfSe and STAMP.

LEfSe	STAMP	LEfSe&STAMP
g__Achromobacter	g__Acinetobacter	g__Acinetobacter
g__Acinetobacter	g__Adhaeribacter	g__Adhaeribacter
g__Adhaeribacter	g__Adlercreutzia	g__Adlercreutzia
g__Adlercreutzia	g__Aeromicrobium	g__Aeromonas
g__Aeromonas	g__Aeromonas	g__Afipia
g__Afipia	g__Afipia	g__Agrobacterium
g__Agrobacterium	g__Agrobacterium	g__Agromyces
g__Agromyces	g__Agromyces	g__Allobaculum
g__Alcanivorax	g__Allobaculum	g__Alteromonas
g__Allobaculum	g__Alteromonas	g__Aquicella
g__Alteromonas	g__Aquicella	g__Arthrobacter
g__Anaerospora	g__Arthrobacter	g__Asticcacaulis
g__Aquicella	g__Asticcacaulis	g__Bacillus
g__Arthrobacter	g__Bacillus	g__Balneimonas
g__Asticcacaulis	g__Balneimonas	g__Bordetella
g__Bacillus	g__Bordetella	g__Bosea
g__Balneimonas	g__Bosea	g__Bradyrhizobium
g__Bordetella	g__Bradyrhizobium	g__Burkholderia
g__Bosea	g__Burkholderia	g__Candidatus Aquiluna
g__Bradyrhizobium	g__Candidatus Aquiluna	g__Candidatus Arthromitus
g__Burkholderia	g__Candidatus Arthromitus	g__Candidatus Koribacter
g__Candidatus Aquiluna	g__Candidatus Koribacter	g__Candidatus Solibacter
g__Candidatus Arthromitus	g__Candidatus Solibacter	g__Cobetia
g__Candidatus Koribacter	g__Cobetia	g__Coccinimonas
g__Candidatus Solibacter	g__Coccinimonas	g__Cupriavidus
g__Chitinophaga	g__Cupriavidus	g__Desulfovibrio
g__Chryseobacterium	g__Desulfovibrio	g__Devosia
g__Cobetia	g__Devosia	g__Dorea
g__Coccinimonas	g__Dorea	g__Duganella
g__Colwellia	g__Duganella	g__Dyella
g__Cupriavidus	g__Dyella	g__Edaphobacter
g__Desulfovibrio	g__Edaphobacter	g__Ensifer
g__Devosia	g__Ensifer	g__Erythrobacter
g__Dorea	g__Erythrobacter	g__Flavisolibacter
g__Duganella	g__Flavisolibacter	g__Flavobacterium
g__Dyella	g__Flavobacterium	g__Glaciecola
g__Edaphobacter	g__Glaciecola	g__Halomonas
g__Ensifer	g__Halomonas	g__Herbaspirillum
g__Erythrobacter	g__Herbaspirillum	g__HTCC

g__Flavisolibacter	g__HTCC	g__Hyphomicrobium
g__Flavobacterium	g__Hyphomicrobium	g__Hyphomonas
g__Glaciecola	g__Hyphomonas	g__Iamia
g__Haliangium	g__Iamia	g__Kaistobacter
g__Halomonas	g__Kaistobacter	g__Kribbella
g__Herbaspirillum	g__Kribbella	g__Lactobacillus
g__HTCC	g__Lactobacillus	g__Lactococcus
g__Hyphomicrobium	g__Lactococcus	g__Lentzea
g__Hyphomonas	g__Lentzea	g__Limnobacter
g__Iamia	g__Limnobacter	g__Loktanella
g__Inquilinus	g__Loktanella	g__Luteibacter
g__Kaistobacter	g__Luteibacter	g__Maricaulis
g__Kribbella	g__Maricaulis	g__Marinobacter
g__Labrenzia	g__Marinobacter	g__Marinomonas
g__Lactococcus	g__Marinomonas	g__Marivita
g__Lentzea	g__Marivita	g__Massilia
g__Limnobacter	g__Massilia	g__Mesorhizobium
g__Loktanella	g__Mesorhizobium	g__Methylibium
g__Luteibacter	g__Methylibium	g__Methylotenera
g__Lysobacter	g__Methylotenera	g__Nautella
g__Maricaulis	g__Mycobacterium	g__Niabella
g__Marinobacter	g__Nautella	g__Nitrosovibrio
g__Marinomonas	g__Niabella	g__Nocardia
g__Marivita	g__Nitrosovibrio	g__Nocardioides
g__Massilia	g__Nitrospira	g__Nonomuraea
g__Mesorhizobium	g__Nocardia	g__Novosphingobium
g__Methylibium	g__Nocardioides	g__Oceanospirillum
g__Methylotenera	g__Nonomuraea	g__Octadecabacter
g__Microbacterium	g__Novosphingobium	g__Olleya
g__Nautella	g__Oceanospirillum	g__Olsenella
g__Niabella	g__Octadecabacter	g__Pedomicrobium
g__Niastella	g__Olleya	g__Pelagibacter
g__Nitrosospira	g__Olsenella	g__Phaeobacter
g__Nitrosovibrio	g__Pedomicrobium	g__Phenylobacterium
g__Nocardia	g__Pelagibacter	g__Phycicoccus
g__Nocardioides	g__Phaeobacter	g__Pilimelia
g__Nonomuraea	g__Phenylobacterium	g__Plesiocystis
g__Novosphingobium	g__Phycicoccus	g__Promicromonospora
g__Oceanospirillum	g__Pilimelia	g__Pseudoalteromonas
g__Ochrobactrum	g__Plesiocystis	g__Psychrobacter
g__Octadecabacter	g__Promicromonospora	g__Ramlibacter
g__Oleispira	g__Pseudoalteromonas	g__Rhizobium

g__Olleya	g__Psychrobacter	g__Rhodanobacter
g__Olsenella	g__Ramlibacter	g__Roseovarius
g__Pedomicrobium	g__Rhizobium	g__Rubrivivax
g__Pelagibacter	g__Rhodanobacter	g__Rubrobacter
g__Phaeobacter	g__Rhodoplanes	g__Shewanella
g__Phaeospirillum	g__Roseovarius	g__Skermanella
g__Phenylobacterium	g__Rubrivivax	g__Sorangium
g__Phycicoccus	g__Rubrobacter	g__Sphingomonas
g__Pilimelia	g__Shewanella	g__Steroidobacter
g__Planktotalea	g__Skermanella	g__Streptomyces
g__Plesiocystis	g__Sorangium	g__Sulfitobacter
g__Polaribacter	g__Sphingomonas	g__Synechococcus
g__Promicromonospora	g__Steroidobacter	g__Tenacibaculum
g__Pseudoalteromonas	g__Streptomyces	g__Thermomonas
g__Psychrobacter	g__Sulfitobacter	g__Tropicibacter
g__Ramlibacter	g__Synechococcus	
g__Rhizobium	g__Tenacibaculum	
g__Rhodanobacter	g__Thalassomonas	
g__Rhodovulum	g__Thermomonas	
g__Roseovarius	g__Tropicibacter	
g__Rubrivivax	g__Vibrio	
g__Rubrobacter		
g__Shewanella		
g__Skermanella		
g__Sorangium		
g__Sphingobacterium		
g__Sphingomonas		
g__Stenotrophomonas		
g__Steroidobacter		
g__Streptomyces		
g__Sulfitobacter		
g__Synechococcus		
g__Tenacibaculum		
g__Thalassospira		
g__Thermomonas		
g__Tropicibacter		
g__Variovorax		
g__Veillonella		
g__Lactobacillus		
